# Relationship of hypomethylation CpG islands in interleukin-6 gene promoter with IL-6 mRNA levels in patients with coronary atherosclerosis

**DOI:** 10.34172/jcvtr.2020.37

**Published:** 2020-09-05

**Authors:** Monireh Mohammadpanah, Mohammad Mehdi Heidari, Mehri Khatami, Mehdi Hadadzadeh

**Affiliations:** ^1^Department of Biology, Faculty of Science, Yazd University, Yazd, Iran; ^2^Department of Cardiac Surgery, Afshar Hospital, Shahid Sadoughi University of Medical Sciences, Yazd, Iran

**Keywords:** Atherosclerosis, Interleukin-6, DNA Methylation, Gene Expression

## Abstract

***Introduction:*** Atherosclerosis is the important cause of most cardiovascular diseases, with high prevalence and mortality. Atherosclerosis is not only a lipid metabolism disorder but also recently is defined as a chronic inflammatory disease. Several studies showed that interleukin-6 (IL-6) is involved in the pathogenesis of atherosclerosis. The aim of the present study is the examination of *IL6* mRNA Levels and hypomethylation of *IL6* promoter in atherosclerosis patients.

***Methods:*** In this assay, a total of 35 cases with atherosclerosis and 30 controls were enrolled. RNA and DNA were isolated from the peripheral blood of all samples. Mean *IL6* gene expression was determined by RT-PCR and methylation status at six CpG motifs in *IL6* promoter was determined using bisulfite genomic sequencing.

***Results:*** Real Time-PCR analysis results showed the mean IL6 RNA level in atherosclerosis patients candidate for CABG (coronary artery bypass grafting) was significantly higher than controls (*P* value = 0.01). Also, the upstream CpG motifs (-1038 to -952) in *IL6* promoter were predominantly unmethylated in patients than in the controls (*P* value = 0.01).

***Conclusion:*** These findings suggest that an increase in *IL-6* gene expression and its DNA hypomethylation promoter are associated with atherosclerosis patient’s candidate for CABG surgery.

## Introduction

Atherosclerosis is the leading cause of cardiovascular disease, including ischemic gangrene, coronary heart diseases, heart failure, abdominal aortic aneurysm, and stroke.^1^ It is responsible for the highest mortality in Western societies, which is increasing significantly in developing countries.^2^ Recent data suggest that the most common risk factor for the adult Iranian population is atherosclerosis.^3-5^ The most important health problem in Iran is the high prevalence and mortality rate.^6,7^

Atherosclerosis develops over a person’s lifetime and involves several steps: Endothelium activation and immune cells employment; the differentiation of monocytes and the formation of foam cell, fibrotic plaques formation due to foam cells death and proliferation and migration of SMCs (smooth muscle cells); and plaque rupture and thrombosis.^2,8^

It has now been well accepted that atherosclerosis is not only a lipid disorder but also chronic inflammatory disease.^9,10^ Inflammation is known as an important factor in the development of atherogenesis through adverse effects on the metabolism of lipoprotein and arterial wall biology. Both innate and acquired immune system has been implicated in the atherogenic process.^9-11^

In atherosclerosis, both immune responses (the innate and adaptive) are organized by a wide range of cytokines that control all of the disease stages. Up to now, a various group of cytokines, low-molecular-weight proteins has been identified. Cytokines are divided into several classes, such as the interleukins (IL), tumor necrosis factors (TNF), chemokines, transforming growth factors (TGF), the interferons (IFN), and colony-stimulating factors (CSF).^8,9,12^ A large body of evidence suggests that interleukin signaling promotes atherogenic activity.^13^

Interleukin-6 is an uncommon cytokine with anti-inﬂammatory and pro-inﬂammatory characteristics, a culprit in the development and complications of atherosclerotic disease.^14^ IL-6 has deep effects on the spread and activation of T cells. The cell differentiation of CD8^+^T cells into cytotoxic T Cells is promoted with IL6^15^ In lymphoid tissue, IL-6 alone or in association with other cytokines stimulates differentiation of T follicular helper (Tfh) cells, T helper 1 and 17.^15-17^

IL-6, together with growth factor (TGF) -β, plays an important role in the differentiation of Th17 cells from naive CD4 + T cells.^15,18,19^ Naive T CD4+ cell precursors are generated Tfh and they participate in antigen-specific B lymphocytes differentiation into memory and plasma cells.^15,20^ Among atherosclerosis patients, the response of systemic inflammatory variability can be explained by different patterns of epigenetic changes that alter the expression of genes involved in the process of the atherosclerotic process.

Mechanisms that modify gene expression without changing the underlying gene sequence are collectively referred to as epigenetic, although that this term is typically used for changes that are heritable through mitosis. Epigenetic changes are sometimes transmitted from parent to child, although it is controversial what role they play in transgenic epigenetic changes in human life. At the DNA level, the underlying mechanisms of short- and long-term regulation of gene expression are overlapped. Epigenetic mechanisms, such as histone modification, DNA methylation, and non-coding RNA can regulate gene expression while the underlying DNA sequence remains the same.^21^ DNA methylation is a significant epigenetic mechanism of the regulation of transcription.^22^ Methylation modification is an alternative mechanism regulating IL-6 production^23,24^ and the consistent evidence has revealed that IL-6 expression is associated with a reduction in the DNA methylation of its gene promoter.^25^

The aim of the current study was firstly, to assay the IL-6 mRNA Levels in the atherosclerosis patients’ peripheral blood, secondly, to examine the methylation status of IL-6 promoter in atherosclerosis patients who are candidate for coronary artery bypass grafting surgery, and finally to evaluate the relationship between methylation status of CpG motifs in IL-6 gene promoter and IL-6 mRNA levels in these patients.

## Materials and Methods

### Study sample

A total of 35 patients with atherosclerosis were recruited from Department of Cardiac Surgery, Afshar Hospital, Yazd, Iran. The sample size was determined at the significance level of α = 0.05 using PASS (Power and Sample Size) software (version 5) and the procedure of comparing the means of two independent groups. The diagnosis of atherosclerosis was established by a cardiologist during the angiographic evidence. The coronary arteries wrap around the entire heart. The left coronary artery (LM) has two main branches called the anterior descending (LAD) and the circumflex (LCX) or rotating artery, and there is a right coronary artery that is called RCA. In a case, if the right coronary artery or one of the branches of the left coronary artery has a blockage of more than 50%, the case is 1VD. If there are two coronary arteries with a blockage of more than 50%, the case is 2VD and the patients who have three coronary arteries with a blockage of more than 50% are 3VD. There was no coronary artery with significant blockage (less than 50%) in control case. As a control group, 30 cardiovascular disease-free subjects, determined by physical examination, history analysis, electrocardiography, and echocardiography were recruited from the same hospital during the same period when the case-patients were recruited. The controls were frequency-matched to the cases by age (±5 years) and sex. For both atherosclerosis and control groups, subjects with hypertension, diabetes, peripheral artery disease, autoimmune-related disease or cancers were excluded. Information on age, sex, height, weight, cigarette smoking, and family history of cardiovascular disease was obtained using a structured questionnaire through in-person interviews. The clinical data and characteristics for the current study are summarized in Table 1. Patients and control cases were divided into four groups: CAD- group with normal coronary artery, and CAD^+^ group with significant lesions (>50% narrowing of luminal diameter) in one, two, or three vessels (LAD, LCX, and RCA). All of the patients were candidates for CABG (Coronary Artery Bypass Grafting) surgery.

**Table 1:** The Summary of the clinical and genetic analysis of coronary atherosclerosis patients

		**Patients**	**Controls**	**χ** ^2^	***P*** **value**
Gender (%)	Male	42.8	40	1.862	0.172
	Female	57.2	60		
Age (%)	<­65	48.2	52.4	1.953	0.179
	>­65	51.8	47.6		
Smoking (%)	Yes	35.9	29.7	1.732	0.192
	No	64.1	70.3		
Family history (%)	Yes	74.2	73.3	1.785	0.189
	No	25.8	26.6		
BMI (kg/m^2^)	-	25.4±1.9	25.1±1.7	**_**	_

### RNA isolation, preparation, and real-time PCR

Total RNA was isolated from whole-blood samples using Total RNA Extraction Kit (Parstous biotechnology, Iran) (Cat No. A101231). The RNA concentration was measured by NanoDrop^TM^ Spectrophotometer (Nanotellectia^TM^ Nano 200, China). Reverse transcription reaction was carried out using the first Strand cDNA Synthesis Kit (CinnaGene, Iran) (CatNo. K1621), according to the manufacturer’s instructions. There was no need to treat the samples with Dnase because the primers in Real-time PCR spanned an exon-exon junction. Real-time PCR was performed with a StepOne^TM^ Real-Time PCR System (Applied Biosystems), using qPCR Master Mix Green-High Rox (Amplicon, England) (Cat. No. A325402). Each PCR reaction consisted of 5 µL of complementary DNA (cDNA), 2 µL of Master Mix (2×), 0.8 µL related primers (0.25 μM), and 2.2 µL of sterile distilled water. Data obtained for the IL6 gene were normalized by comparison with HPRT1 gene expression. The primer sets were designed based on the sequences from the NCBI database. The primers for IL-6 (GenBank Accession No. XM_011515390.2) were 5′-GGTACATCCTCGACGGCATC-3′ (forward) and 5′-GTGCCTCTTTGCTGCTTTCAC-3′ (reverse), and the primers for HPRT1 (GenBank Accession NM_000194.3) were 5’-TATTCCTCATGGACTAATTATGG-3’ (forward) and 5′- CCTCCCATCTCCTTCATCAC -3′ (reverse). The assay was performed under the following condition: initial denaturation stage at 95°C for 15 minutes, followed by 40 cycles with denaturation at 95°C for 15 seconds, annealing at 59.5°C for 15 seconds, and extension at 72°C for 20 seconds. Relative Quantification (RQ) was calculated using the comparative CT.

### Bisulfite genomic sequencing

Genomic DNA was extracted using the DNeasy Blood and tissue kit (QIAGEN) (Cat No./ID: 69504) following the manufacturer’s protocol. The whole blood genomic DNA (500 ng) was converted by sodium bisulfite according to the manufacturer’s instructions of the *EpiTect Fast DNA Bisulfite* Kit (QIAGEN) (Cat No./ID: 59824). This kit transforms unmethylated cytosine into uracil without changing methylated cytosine.^26^

Touchdown PCR was performed in a 20 μL reaction volume containing 7 μL converted DNA by sodium bisulfite, 10 μL of PCR Master mix (2×), 2 μL of forward and reverse primers (2.5 mM), and 1 μL sterile distilled water. The Touchdown PCR was done under the following condition: initial denaturation stage at 95°C for 5 min, followed by 6 cycles of 95°C for 1 minute, 55- 58°C for 50 seconds, and 72°C for 50 seconds, followed by 35 cycles of 95°C for 1 minute, 53°C for 50 seconds, 72°C for 50 seconds, and final extension at 72°C for 5 minutes. The forward and reverse primers for this PCR reaction were 5’-GGTTTTTGAATTAGTTTGATT-3’ and 5-CCCTATAAATCTTGATTTAAAAT-3’ respectively.^27^

For the detection of PCR products, 2% agarose gel and staining with ethidium bromide were used. Methylation level was expressed as methylated cytosine percent on total methylated and non-methylene cytosines. Non-CpG cytosine residues are used as built-in controls to check bisulfite conversion. The values were expressed as the mean for all the sites and separately for six CpGs at the*IL-6* gene promoter.

### Statistical analysis

Variables were tested in triplicate, and experiments were repeated at least twice. Z-score standardized values were used for both mean of CpG island methylation values and 2-^∆Ct^, excluding outliers from analyses. Pearson correlation and t Student test were carried out using (SPSS) software (IBM SPSS 22, SPSS Inc., Chicago, IL., USA) and values of *P <* 0.05 were considered statistically significant.^28^

### Bioinformatics analysis

The nucleic acid sequence was investigated from the National Centre for Biotechnology Information (https://www.ncbi.nlm.nih.gov/).

The sequence alignment was performed using the multiple sequence alignment software MEGA6. UCSC Genome Browser (https://genome.ucsc.edu/) was used to determine the intrinsic properties of promoters, such as the methylation status of CpG islands, sequence conservation, and examining DNA sensitivity to the enzymes DNase. To annotate regulatory sites in the 5′-UTR region of the IL-6 gene, Swissregulon, an online portal, was applied (http://swissregulon.unibas.ch/sr/). Other databases for exploration and visualization of predicted transcription factor binding sites (TFBSs) were PROMO (http://alggen.lsi.upc.es/cgi-bin/promo_v3/promo/promoinit.cgi?dirDB=TF_8.3), Sabiocsience (http://gncpro.sabiosciences.com/gncpro/gncpro.php), ChIP array (http://www.jjwanglab.org/) and PReMod (https://omictools.com/premod-tool).

## Results

The chi-square test was performed for each trait between the two patients and control groups. At first, the percentages for all the studied traits were calculated in two groups of patients and normal cases, and it was evaluated the qualitative variables in both groups. The chi-square and the *P* values were displayed in each comparison in Table 1. *P* values for all of them were more than 0.05. They ​​indicate that there is no significant difference between the two groups in terms of gender, age, sex, smoking, and family history of cardiovascular disease. Therefore, the selection of samples for this study was done correctly and the involvement of other factors such as these has been minimized in the results of the study.

The mRNA expression levels of different genes in patients with atherosclerosis and healthy subjects were determined by RT-qPCR technique. The quantitative data of IL6 and HPRT1 genes expression by the RT-PCR were also analyzed by SPSS software. To do this, after calculating the mean and standard deviation, *t* test was performed on the data (Table 2). The t-test is used to compare the mean of two independent groups and it proves statistically the existence or absence of a significant difference between the two groups. In the first part of the Table 2, the results of the Levene’s test can be seen. This test shows whether the variance of the IL-6 gene expression for the two groups (patient and control) is the same or not. The results of the Levene’s test determine which SPSS outputs will be correct for use.

**Table 2:** The results of *t* test and the comparison of patients and control in IL6 gene expression

**Independent Samples Test for IL6 Gene Expression**					
	**Levene's test for equality of variances**		**T-Test for equality of mean**		
	**F**	**Sig.**	**T**	**df**	**Sig (2-tailed)**
Equal Variances assumed	11.208	0.167	15.983	63	0.01

If the value of Sig. in Levene’s test be greater than 0.05, the data in the first row of the table must be used, because they indicate that the variances of the two study groups are the same (Equal variance assumed). While if the value of Sig. be less than 0.05, it means that the variances are not the same and the data in the second row of the table (Equal variance not assumed) must be used.

As it is seen in Table 2, the value of Sig. in the Levene’s test is equal to 0.167. This indicates the same variances in IL-6 gene expression in both the patient and control groups. In other words, the two groups are homogeneous, and each on follows a normal distribution curve. Therefore, the conclusion of this data can be reliable.

**Figure 1:**
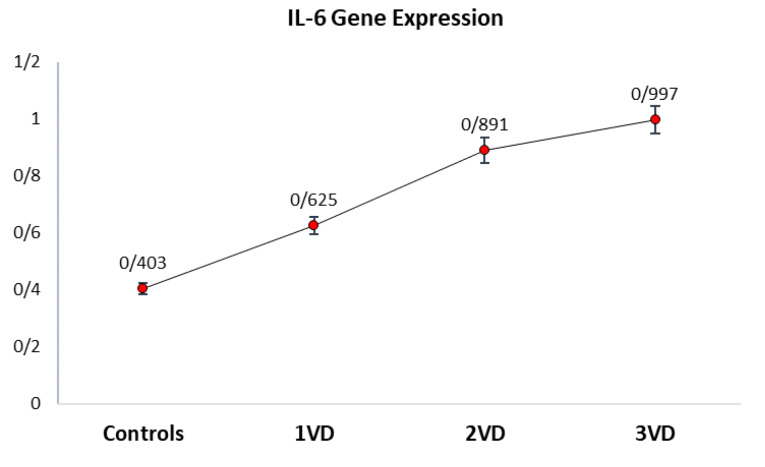
Comparison of mean IL-6 mRNA expression levels measured in peripheral blood in atherosclerosis patients (3VD, 2VD, and 1VD) and controls. The Real-time assay showed that the average IL-6 gene expression levels were different in 1VD, 2VD, 3VD, and controls. It illustrates that the average of IL-6 gene expression in 3VD patients is the highest, and it is the lowest in control cases

The qPCR analysis showed that the*IL-6* mRNA expression level in patients with atherosclerosis was significantly higher than that in controls (*P* < 0.05). Among those who had coronary plaques, patients with 3VD, 2VD, and 1VD showed more levels of the IL-6 mRNA expression in whole blood than controls, respectively. Figure 1 illustrates that the average of IL-6 gene expression in 3VD patients is the highest, and it is the lowest in controls.

**Figure 2:**
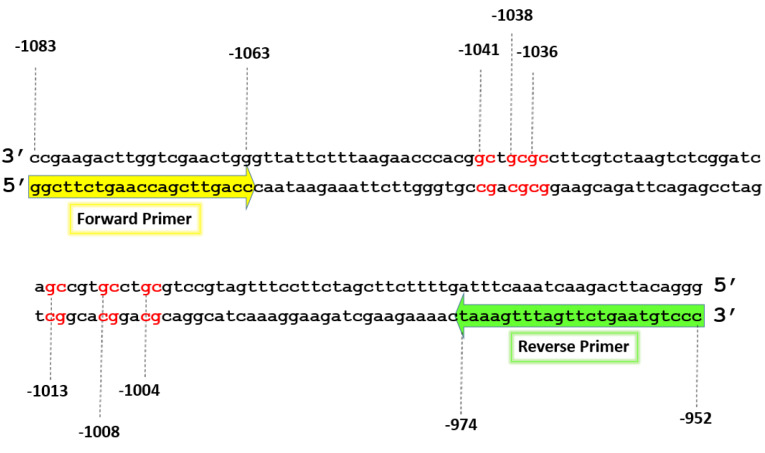
The 6 CpG motifs of the IL-6 gene promoter from region –1083 bp to -952 bp

**Figure 3:**
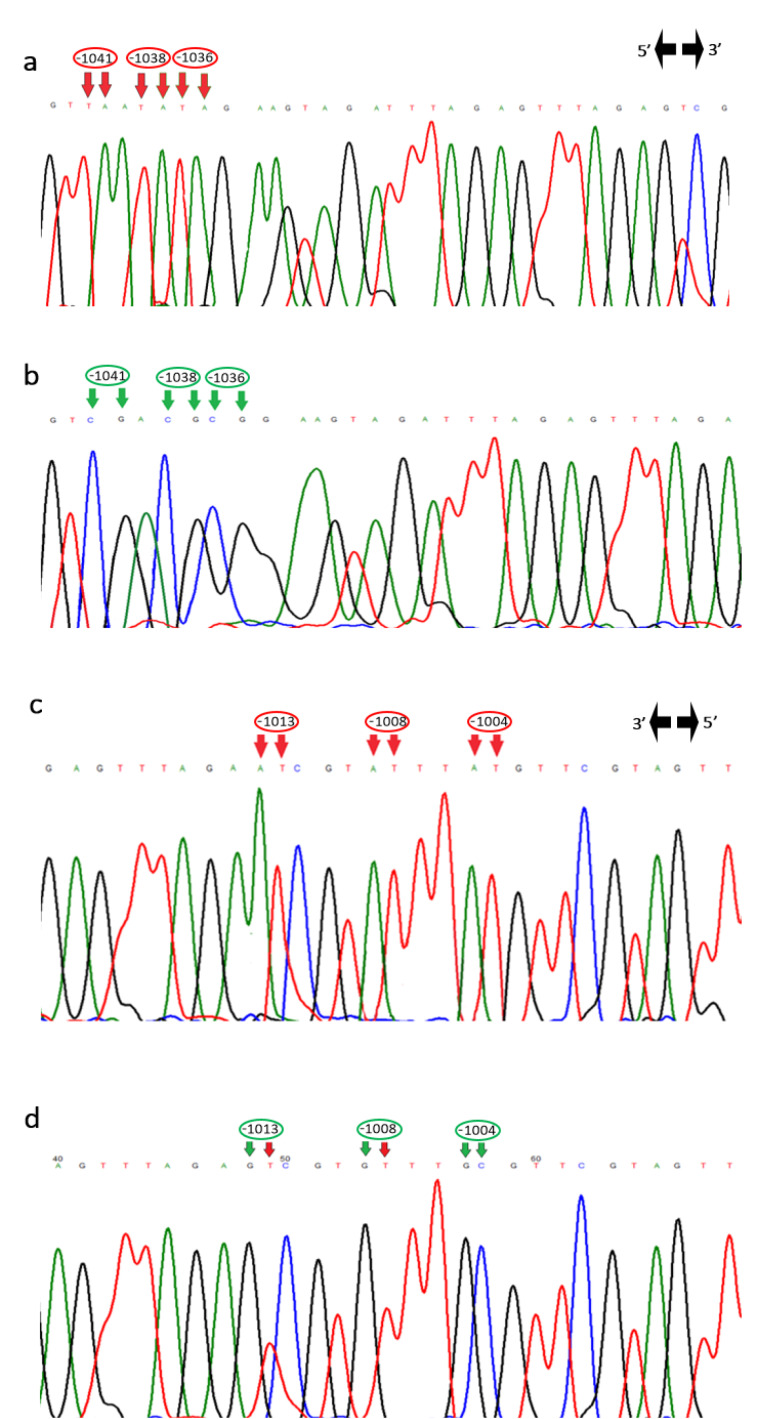
DNA sequencing electropherograms show the CpG motifs at -1041C, -1038C, and -1036C were unmethylated in one patient’s sample (a) and methylated in control (b). The CpG motifs at -1013C, -1008C, and -1004C were also unmethylated in one patient’s sample (c) and methylated or semi-methylated in control (d)

IL-6 gene expression level was also associated with its DNA methylation pattern. The region of the IL-6 gene promoter from nucleotide -1083 to -952 contains 6 CpG motifs (Figure 2). The methylation status of this region was compared in patients with atherosclerosis and control groups. According to bisulfite sequencing results (Figure 3), the six CpG motifs in this region were predominantly methylated or semi-methylated in controls, while CpG motifs at -1041C, -1038C, and -1036C in patient group were predominantly unmethylated (*P* < 0.05), and the CpG motifs at -1013C, -1008C, and -1004C were methylated (*P* > 0.05).

**Figure 4:**
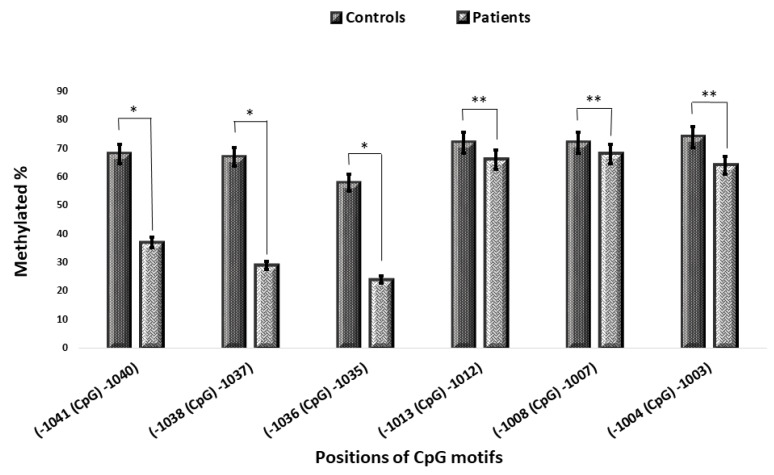
Comparison of the methylation of specific CpG motifs in pripheral blood from patients with atherosclerosis and healthy controls. The methylation status of CpG motifs at -1041C, -1038C, and -1036C presented a significant difference in patients and controls, while there is no significant difference in the methylation status of the other CpG motifs at -1013C, -1008C, and -1004C in both groups (*P -value < 0.05, **P -value > 0.05)

**Figure 5:**
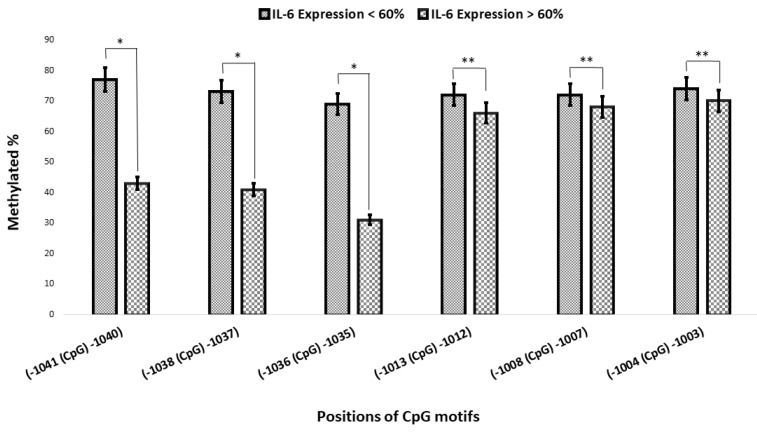
Comparison of the methylation of CpG motifs with IL-6 mRNA level. The methylation status of CpG motifs at –1041C, -1038C, and -1036C presented a significant difference in population with higher levels of IL-6 gene expression and population with lower levels (*P -value < 0.05, **P -value > 0.05).

Two arbitrary groups were defined according to the mean levels of IL-6 mRNA expression, and the methylation of the 6 CpG motifs between -1083 and -952 was compared in both healthy controls and atherosclerotic patients. The results were similar to those obtained previously (Figure 4). The CpG motifs at -1041C, -1038C, and -1036C were predominantly methylated in cases showed lower IL-6 mRNA expression levels (controls and some of 1VD patients), while these motifs were unmethylated or semimethylated in cases with higher levels of mRNA expression (2VD and 3VD patients), and it showed a significant difference (*P* < 0.05).^29^ The status of methylation in CpG motifs at -1013C, -1008C, and -1004C was similar and both arbitrary groups were methylated (*P* > 0.05) (Figure 5).

The correlation between methylation and gene expression level in the two groups of patients and control was measured by Pearson correlation coefficient test. The results of this test are shown in Table 3. According to it, the correlations between positions -1041C, -1038C, and -1036C and gene IL6 mRNA levels are negative in patients and positive in control cases. The p-values prove the correlations are significant in both groups (correlation is significant at Sig. less than 0.05). Therefore, by increasing the expression of IL6 gene, all these three positions have shown a decrease in methylation status in patients, and by decreasing the IL6 mRNA levels, all these three positions have indicated an increase in methylation status in controls.

**Table 3:** The results of Pearson Correlation coefficient test and the investigating the correlation between IL6 gene expression and methylation status

**Pearson correlation coefficient**		
		**IL6 gene expression**
Patients	-1041 (CpG) -1040 Pearson correlation	-0.719
	Sig. (2-tailed)	0.01
	-1038 (CpG) -1037 Pearson correlation	-0.821
	Sig. (2-tailed)	0.01
	-1036 (CpG) -1035 Pearson correlation	-0.786
	Sig. (2-tailed)	0.01
	-1013 (CpG) -1012 Pearson correlation	0.759
	Sig. (2-tailed)	0.5
	-1008 (CpG) -1007 Pearson correlation	0.814
	Sig. (2-tailed)	0.5
	-1004 (CpG) -1003 Pearson correlation	0.901
	Sig. (2-tailed)	0.5
Controls	-1041 (CpG) -1040 Pearson correlation	-0.761
	Sig. (2-tailed)	0.01
	-1038 (CpG) -1037 Pearson correlation	-0.813
	Sig. (2-tailed)	0.01
	-1036 (CpG) -1035 Pearson correlation	-0.891
	Sig. (2-tailed)	0.01
	-1013 (CpG) -1012 Pearson correlation	-0.921
	Sig. (2-tailed)	0.5
	-1008 (CpG) -1007 Pearson correlation	-0.891
	Sig. (2-tailed)	0.6
	-1004 (CpG) -1003 Pearson correlation	-0.789
	Sig. (2-tailed)	0.5

The correlations between methylation status of positions -1013C, -1008C, and -1004C and gene expression in the two studied group were not significant (correlation is not significant at Sig. more than 0.05).

The results of sequence alignment of IL-6 gene among numerous species presented that these six CpG motifs were located in highly conserved regions among several species (human, gibbon, cow, chimpanzee, elephant, mouse, dog, rat, chicken, and zebrafish).

## Discussion

Interleukin-6, a pleiotropic cytokine, regulates a variety of inflammatory responses. The formation of atherosclerotic plaques is caused by the accumulation of many genetic and epigenetic molecular changes.

Previous studies were shown that IL-6 level is raised in CAD patients and may be an inﬂammation marker for cardiovascular risk.^30^ The results of Lefkou et al displayed that children (5-15 years) with a family history of premature coronary artery disease meaningfully increased IL-6 plasma levels compared to the control group of pediatric cases without a family history (*P* < 0.001).^31^

The findings of Fisman et al showed that people with stable coronary artery disease had higher IL-6 with a weaker prognosis over an average follow-up of about 6 years. In their study, the increase of IL-6 (each 1 pg/mL) was associated with increased relative odds 1.70 (range 1.23-2.45) of following sudden death.^32^

In research in China, with 263 patients with ST-segment elevation myocardial infarction, the admission level of IL-6 was correlated with cardiovascular mortality for more than 3 years of follow-up.^33^ Additionally, Murine experiments, with a number suggest that they do not protect the infarction damage with the elimination of IL-6 effects.^34^

However, there is little information about the profile of CpG spots and their methylation status in the promoter of the IL-6 gene in patients with atherosclerosis. In our study, a total of six CpG motifs in the IL-6 promoter was determined using bisulfite genomic sequencing in the Iranian population.

An active process is DNA methylation that catalyzed by DNA methyltransferases in cytosine residues in CpG dinucleotides. The previous studies suggested that the expression of these enzymes was influenced by several factors including cigarette age, sex, and smoking. These studies show that the mRNA level of DNA methyltransferase was higher in smokers than in nonsmokers. Also, in human cells and tissues, an age-dependent decrease of the methylation levels of DNA cytosine-5-methyltransferase and methylation was shown.^35^ The findings of Zuo suggested that DNA hypomethylation of IL-6 promoter is associated with the increased risk for acute myocardial infarction.

According to the results of the transcription regulation database to identify transcription factors DNA binding to these six CpG motifs, the importance of these CpG motifs are highlighted for controlling transcription rate and gene expression (Table 4). The methylated or unmethylated status will affect the binding of transcription factors to the DNA sequence. The status of DNA methylation is lower in transcriptionally active genes, and our results are consistent with the concept that methylation is important in controlling the expression of IL-6 as seen in atherosclerosis. All of the participants in this study were exclusive of the average age of the elderly, so the generalization of our findings to young people or other races/ethnic groups is uncertain. We measured only interleukin-6 with expression levels from blood taken during a physical examination, but the IL-6 concentration may change over time. It should be pointed out that our findings do not prove a direct causal relationship between IL-6 promoter hypomethylation and atherogenesis, but just describe an association between these two processes.

**Table 4:** Transcription Factorsbinding to DNA sequence andCpG motifs according to Swissregulon, Sabiocsience, PROMO, PReMod, and ChIP array data base

**CpG motif**	**Transcription Factors**
-1041 (CpG) -1040	RXR-alpha [T01345]
-1038 (CpG) -1037	c-Ets-1 [T00112] and Elk-1 [T00250]
-1036 (CpG) -1035	STAT4 [T01577], c-Ets-1 [T00112], E2F-1 [T01542], and Elk-1 [T00250]
-1013 (CpG) -1012	STAT4 [T01577], c-Ets-1 [T00112], Elk-1 [T00250], and E2F-1 [T01542]
-1008 (CpG) -1007	STAT4 [T01577] and E2F-1 [T01542]
-1004 (CpG) -1003	GR-alpha [T00337], AP-2alphaA [T00035], and XBP-1 [T00902]

## Conclusion

Our study reported a significant increase in IL-6 gene expression in whole blood in patients with atherosclerosis than controls. These findings suggest that DNA hypomethylation of IL-6 gene promoter and increased mean IL-6 gene expression are associated with the risk of atherosclerosis. Other mechanisms might be involved in the regulation of gene expression that needs further investigations. To confirm the causal relationship between TF-DNA binding and methylation, additional laboratorytests such as analyzing motif mutationsin specific loci and assessing their impact on the local DNA methylation changes are needed.

## Competing interests

None declared.

## Acknowledgments

This research was funded by Yazd University. We thank all the patients for providing blood samples for scientific research, also, the Especial Afshar Hospital (Yazd, Iran).

## Ethical approval

All procedures performed in studies involving human participants were in accordance with the ethical standards of the institutional and/or national research committee and with the 1964 Helsinki Declaration and its later amendments or comparable ethical standards. Informed consent was obtained from all individual participants involved in the study. The study protocol was approved by the local ethics committee of the Yazd University (Code: IR.SSU.REC.1395.223).

## Funding

This project has been done by a grant No. 8923 of Yazd University.
